# Supplementary material for: Learning curve and surgical outcome of robotic assisted colorectal surgery with ERAS program
Source: Sci Rep. 2022 Nov 29;12:20566. doi: 10.1038/s41598-022-24665-w (PMC9709162; doi:10.1038/s41598-022-24665-w)
Supplement: Supplementary file 1 — Supplementary Information 1. [file 41598_2022_24665_MOESM1_ESM.zip › LSC Raw data-Ñ[▒K/077/N_ñw╝╨░O▒Kñσ.pdf]

W83 / W83

臺中榮民總醫院
手術室護理記錄

[redacted]

基本資料

- 室別： 二醫手術室-32室，手術科別： CRS，手術類別： 常規，日期： 2020/04/22
- 進入時間： 1055 ； 麻醉開始時間： 1100 ； Time Out時間： 1148 ； 劃刀時間： 1149 ； 手術結束時間： 1510 送出時間： 1515 ；
- 麻醉方式： GE

  診斷：Malignant tumor of sigmoid colon 20 cm FAV

  手術名稱：Laparoscopic Anterior resection and anastomosis - malignant

  手術局部用藥：nil

  術前預防性抗生素：
  - 抗生素名稱一： Veterin 1GM IVD
  - 時間： 1110
  - 上次簽章: 梁凱雯

  ，追加預防性抗生素：
- 手術醫師： 林俊余，林昌霖，王郁棋
- 麻醉醫師： VS 施涵薰
-
  - 刷手護士A ： 謝昀安，時間： 1055-1200，刷手護士B ： 蔡易軒，時間： 1055-1200，接替刷手一： 顏育?，時間： 1200-1230，接替刷手二： 謝昀安，時間： 1230
  - 流動護士A ： 詹佳柔，時間： 1055-1200，接替流動一： 梁凱雯，時間： 1200-1230，接替流動二： 詹佳柔，時間： 1230
- 更多接替及流動護士： 接替刷手三： 蔡易軒，時間： 1230，接替流動三： 謝昀安，時間： 1500

皮膚狀況

A-V Shunt： 無

術前： 完整

記錄者： 上次簽章: 梁凱雯

術後： 完整

記錄者： 謝昀安

手術準備

手術臥式 單· ·手術臥式

-
  - 手術臥式： 截石臥式
  - 使用附件： 溫毯 38 ℃，頭圈，腳架
  - 皮膚準備： 10%Aq Betadine，Hibiscrub，其它： 2%chlorhexidine
  - 電燒： 使用記錄者： 上次簽章: 梁凱雯

止血帶T： 無

植入物

- 植入物名稱/號碼/數量/部位：HYALOBARRIER GEL 10ML/PELVIC

，記錄者： 上次簽章: 詹佳柔

標本送檢

- 細菌培養 無
- 細胞學 無
- 冰凍切片 無
- 病理標本

  組織名稱一：S COLON

  ，

  組織名稱二：IMA ROOT

  ，

  組織名稱三：DISTAL CUT END

- 截肢 無

標本交付 標本： 3 袋 ， 標本： 0 盒 ， 標本： 0 瓶 ， 細胞學： 0 管 ， Culture Tube： 0 管 ， 被交付醫師： CR 林昌霖 ，

- 記錄者： 謝昀安
- 時間： 1505

傷口術後敷料

傷口敷料：　　傷口敷料： 無

- 請於圖上標示部位：

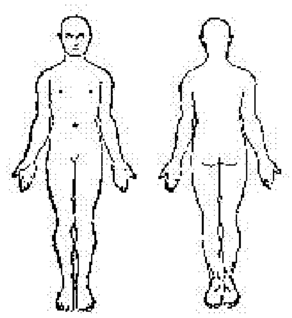

腹部腹腔鏡肆切口

- 傷口敷料： mepore

導管

導管	天數	導管資訊	插入日期	插入時間	更換日期	拔除日期	拔除時間	拔除原因	預計拔除/更換	預計拔除/更換日期
Foley(1)	1	號碼:16 Fr.; 部位:膀胱;	2020/04/22	1115						
Penrose Drain(1)	1	號碼:12 mm.; 部位:骨盆腔; 註記:左下腹;	2020/04/22	1400						
Sump NG Tube(1)	1	號碼:16 Fr.; 部位:胃;	2020/04/22	1115						

用物計數

- 手術中出血量： MINIMAL c.c.
- 手術中尿量： 300 c.c.
-
    - 其它： 無

用物計數： 多切口同時縫合

- 1.器械： 正確 ，2.縫針： 正確 ，3.刀片 ： 正確 ，4.紗布： 正確 ，5.腹部墊： 未使用 ，6.紗球： 未使用 ，7.PEANUTS ： 未使用 ，8.紗條： 未使用 ，9.絲帶： 未使用 ，10.針頭： 未使用 ，11.Vessel loop： 未使用 ，12.Boots： 未使用 ，13.Jelco： 未使用 ，14.棉條： 未使用 ，
    - 第一次：
        - 刷手護士 ： 蔡易軒/謝昀安
        - 流動護士： 上次簽章: 詹佳柔
        - 時間： 1402

,
    - 第二次：
        - 刷手護士 ： 蔡易軒/謝昀安
        - 流動護士： 上次簽章: 詹佳柔
        - 時間： 1417

,
    - 第三次：
        - 刷手護士 ： 蔡易軒/謝昀安
        - 流動護士： 上次簽章: 詹佳柔
        - 時間： 1430

備註

備註： NIL

- 記錄者： 謝昀安
- 時間： 1515

填寫護理人員 謝昀安

正式摘要

		W83 / W83

doctorRank:B
